# A nomogram model to predict recurrence of early-onset endometrial cancer after resection based on clinical parameters and immunohistochemical markers: a multi-institutional study

**DOI:** 10.3389/fonc.2024.1442489

**Published:** 2024-11-11

**Authors:** Yunfeng Zheng, Qingyu Shen, Fan Yang, Jinyu Wang, Qian Zhou, Ran Hu, Peng Jiang, Rui Yuan

**Affiliations:** ^1^ Department of Gynecology, The First Affiliated Hospital of Chongqing Medical University, Chongqing, China; ^2^ Department of Gynecology, Chongqing Yubei Maternity & Child Healthcare Hospital, Chongqing, China; ^3^ Centre for Lipid Research & Chongqing Key Laboratory of Metabolism on Lipid and Glucose, Key Laboratory of Molecular Biology for Infectious Diseases (Ministry of Education), Department of Infectious Diseases, The Second Affiliated Hospital, Chongqing Medical University, Chongqing, China

**Keywords:** early-onset endometrial cancer, traditional clinical parameters, immunohistochemical makers, nomogram, recurrence, risk stratification

## Abstract

**Objective:**

This study aimed to investigate the prognosis value of the clinical parameters and immunohistochemical markers of patients with early-onset endometrial cancer (EC) and establish a nomogram to accurately predict recurrence-free survival (RFS) of early-onset EC after resection.

**Methods:**

A training dataset containing 458 patients and an independent testing dataset consisting of 170 patients were employed in this retrospective study. The independent risk factors related to RFS were confirmed using Cox regression models. A nomogram model was established to predict RFS at 3 and 5 years post-hysterectomy. The C-index, area under the curve (AUC) of the receiver operating characteristic (ROC) curve, and calibration curve were calculated to assess the predictive accuracy of the nomogram.

**Results:**

In all early-onset EC patients, more than half (368/628, 58.6%) were diagnosed in the age range of 45-49 years. Meanwhile, the recurrence rate of early-onset EC is approximately 10.8%. Multivariate Cox regression analyses showed that histological subtype, FIGO stage, myometrial invasion, lymphovascular space invasion (LVSI), P53 expression, and MMR status were independent prognostic factors related to RFS (all *P* < 0.05) and established the nomogram predicting 3- and 5-year RFS. The C-index and calibration curves of the nomogram demonstrated a close correlation between predicted and actual RFS. Patients were divided into high- and low-risk groups according to the model of RFS.

**Conclusions:**

Combining clinical parameters and immunohistochemical markers, we developed a robust nomogram to predict RFS after surgery for early-onset EC patients. This nomogram can predict prognosis well and guide treatment decisions.

## 1 Introduction

Endometrial cancer (EC) is the most prevalent gynecological malignancy of the female reproductive system (1, 2). The incidence rate of EC is increasing annually, and due to lifestyle changes, its population becomes progressively younger. The average age at diagnosis for EC patients is 61 years, with postmenopausal vaginal bleeding being the most common clinical presentation, accelerating the early diagnosis of EC (3). However, the presence of atypical symptoms such as menstrual irregularities could mask the condition, making early diagnosis challenging for young EC patients.

Early-onset EC refers to individuals under 50 years diagnosed with EC (4, 5). Previous studies have shown that compared to late-onset EC (age ≥ 50 years), patients with early-onset EC have better tumor differentiation and prognosis (6). However, recurrence remains a leading cause of reduction in survival rates among this specific group, with a five-year overall survival from 15% to 17% (7). This highlights the imminent need to develop novel assessment modalities to predict recurrence of EC and improve the prognosis outcomes for this population of patients. Recently, the Cancer Genome Atlas (TCGA) Research Project unveiled a genomic reclassification of EC with four distinct subtypes: DNA polymerase epsilon catalytic subunit (*POLE*) ultramutated, microsatellite instability (MSI), copy-number low (CNL) and cope-number high (CNH) (7, 8). The molecular subtype demonstrates good prognostic value for EC patients, but its high cost of detection and demanding requirements for equipment and detection technology levels make it challenging to be widely promoted and applied in many regions and countries in a short period of time. Immunohistochemistry remains a fast, cost-effective, and reliable postoperative assessment method, allowing it to be easily integrated into the clinical management of EC and widely used in clinical practice.

Currently, the management of young patients, especially the risk assessment of recurrence in early-onset EC patients, is a contentious issue. Therefore, a comprehensive consideration of clinical features and immunohistochemical markers to understand the main factors influencing the recurrence-free survival (RFS) of early-onset EC patient could help to predict, prevent recurrence, or at least improve therapy.

Herein, we analyzed 628 early-onset EC patients from multiple centers to identify independent prognosis factors associated with RFS. Subsequently, we established a nomogram model to accurately predict RFS, allowing patients to receive earlier prognosis information, and facilitating optimization of treatment planning.

## 2 Materials and methods

### 2.1 Patients and database

This retrospective large-sample multicenter study included the First Affiliated Hospital of Chongqing Medical University (FAHCQMU, n = 458 cases), Women and Children’s Hospital of Chongqing Medical University (WCHCQMU, n = 140 cases), and Chongqing Yubei District Maternal and Child Health Center (CYMCHC, n = 30 cases). A total of 628 patients who diagnosed with early-onset EC in three medical centers from October 20, 2014 to May 20, 2021 were included.

**Figure 1:**
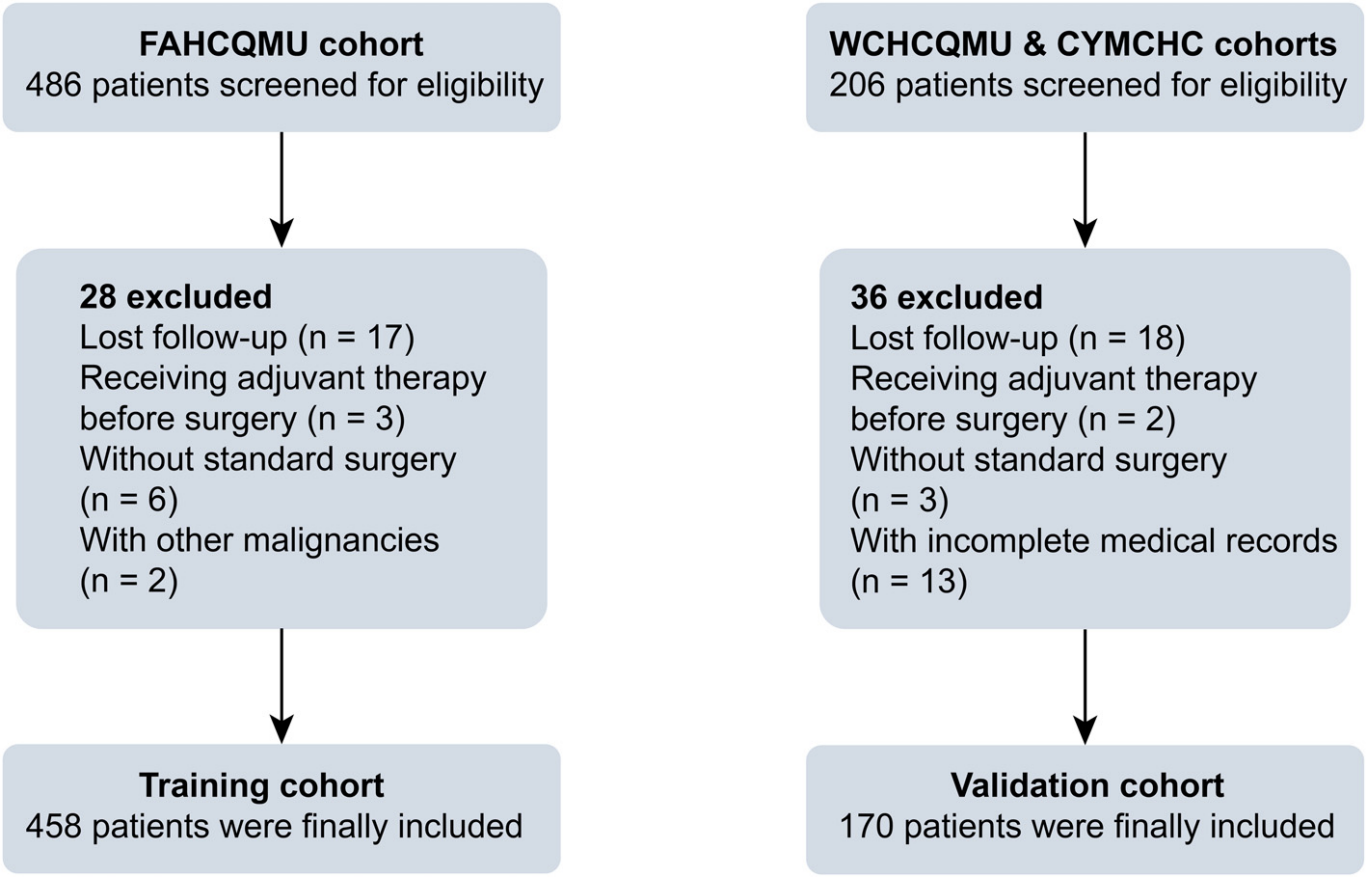
Flow chart for early-onset EC patient inclusion.

The inclusion criteria were as follows: (1) primary EC patients with age less than 50 years; (2) no other malignant tumors; (3) comprehensive clinical and postoperative pathological information. The criteria for exclusion were as follows: (1) patients with age ≥ 50 years old; (2) administration of preoperative adjuvant therapy; (3) incomplete medical records; (4) no standard surgical treatment; (5) loss to follow-up after surgery. The schematic representation of the study flow is illustrated in **Figure 1**.

### 2.2 Surgical procedures and postoperative adjuvant treatment

All patients underwent a surgical staging procedure, including total hysterectomy, bilateral salpingo-oophorectomy, with or without lymph node staging (including sentinel lymph node ± pelvic lymph node ± para-aortic lymphadenectomy) (2, 9). After comprehensive surgery and pathological staging, EC patients will be advised to consider observation or various postoperative adjuvant treatments based on international guidelines and the assessment of risk factors (10, 11). When molecular classification is known, specific recommendations exist for *POLE* mutations (*POLE*mut) and P53 abnormal (P53abn) (see ESGO/ESTRO/ESP Guidelines) (12). Radiotherapy mainly consisted of vaginal brachytherapy (total radiation dose ranges from 22 to 24 Gy, administered in 4 fractions of 5.5-6 Gy, 2 fractions per week) or pelvic external beam radiotherapy total radiation dose ranges from 45 to 50 Gy, administered in 25 fractions of 1.8-2 Gy, 5 fractions per week), administered within 12 weeks post-operative. The chemotherapy regimen is mainly based on the TP regimen (carboplatin combined with paclitaxel) administered every 3 weeks for 6-8 cycles (13).

### 2.3 Patients characteristics collection and follow-up investigation

Clinical pathological and follow-up data were collected by well-trained assistants and recorded using standardized data collection forms, and reviewed by a senior doctor. Specifically, clinicopathological data and surgical details were meticulously collected from medical records, including age at diagnosis, body mass index (BMI), International Federation of Gynecology and Obstetrics (FIGO) stage, histological subtype (categorized as type I or type II), depth of myometrial invasion (categorized as <1/2 or ≥1/2), cervical stromal invasion, lymphovascular space invasion (LVSI, categorized as none, focal, or substantial LVSI) (**Supplementary Table S1**) (14, 15). In the present study, the optimal age cut-off point of early-onset EC was determined based on RFS using X-tile software (**Supplementary Figure S1**) (16). The follow-up was performed every 3 months for the first 2 years, every 6 months for the next 3-5 years, and once per year thereafter.

### 2.4 Recurrence

Recurrences were diagnosed by physical examination and/or imaging, and were confirmed histologically. RFS was calculated as the duration from the initial surgery to the confirmation of the first recurrence or the last follow-up date, with the follow-up deadline scheduled for July 19, 2024.

### 2.5 Histology, MMR and P53 immunohistochemistry

**Figure 2:**
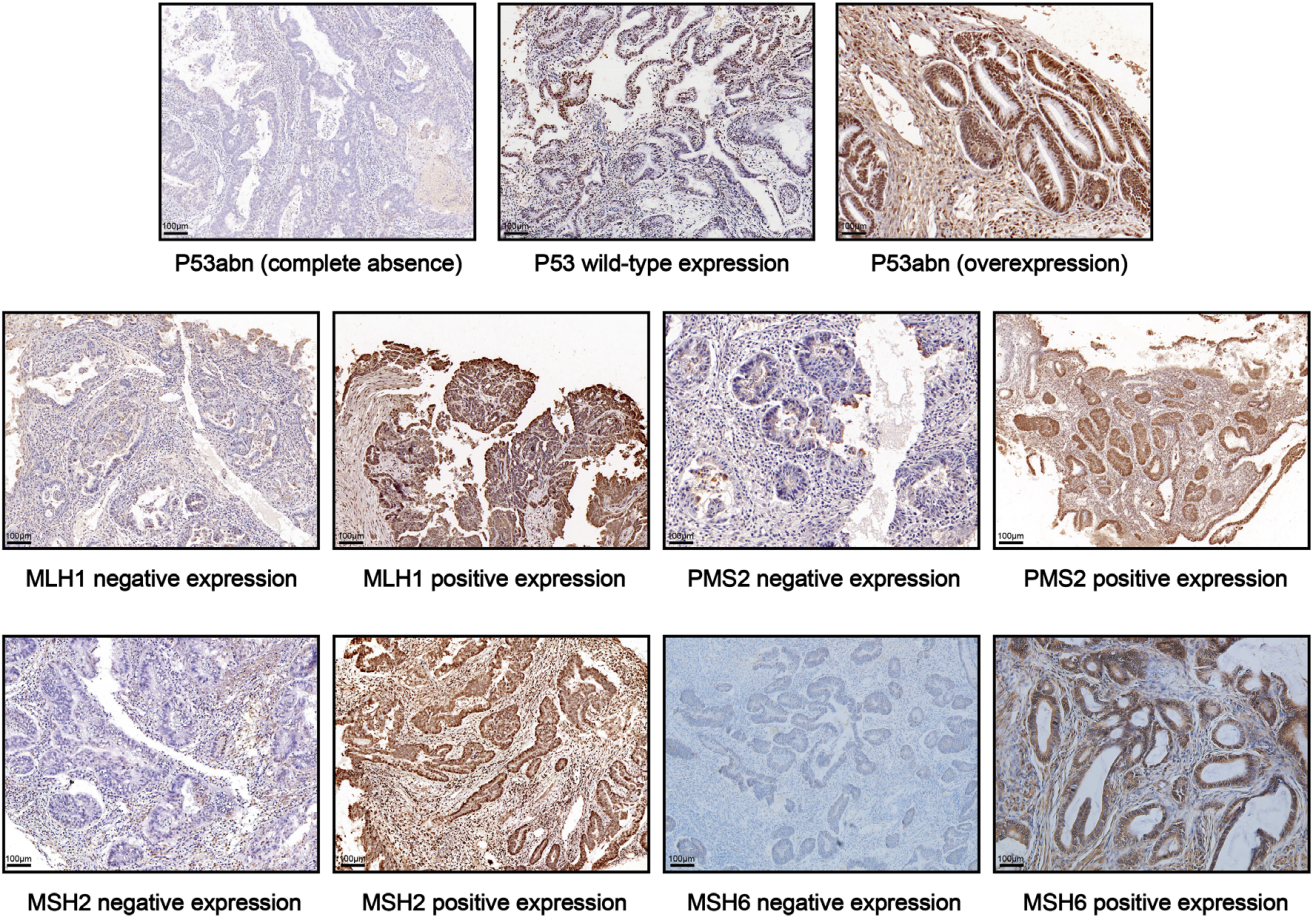
Representative IHC images of P53 and MMR protein expression in EC tissues.

Immunohistochemistry (IHC) was performed on formalin-fixed paraffin-embedded specimens. All pathology slides underwent analysis by experienced pathologists. P53 IHC results were categorized as normal (1-80% of tumor cell nuclei staining positive), or abnormal (no tumor cell nuclear staining; at least 80% of tumor cell nuclei staining positive) (17). The loss of nuclear expression of at least one mismatch repair (MMR) proteins (MLH1, MSH2, MSH6, and PMS2) was defined as MMR-deficiency (dMMR); and positive nuclear staining of all four MMR proteins was defined as proficient MMR (pMMR) (18–20). Representative images of IHC staining for P53 and MMR proteins in early-onset EC tissues are shown in **Figure 2**.

### 2.6 Statistical analysis

Statistical analysis was performed using R software (version 4.0.3) and SPSS software (version 22.0). Categorical data were presented as frequencies with percentages, and statistical comparison for categorical variables were calculated using the χ^2^ or Fisher exact test. Continuous variables were reported as means with standard deviations, and compared using a two-tailed Student’s t test (21). RFS probabilities were estimated using the Kaplan-Meier curves, and group comparisons were analyzed by the log-rank test. Patients from FAHCQMU cohort were included in developing the nomogram model, while patients from WCHCQMU & CYMCHC cohorts were enrolled as the external validation cohort. X-tile software (version 3.6.1) was utilized to determine the diagnostic thresholds for nomogram scores. Subsequently, patients were divided into low- and high-risk groups based on the risk score threshold (16). *P*-values < 0.05 were considered as statistically significant.

## 3 Results

### 3.1 Demographic and clinical characteristics

A total of 628 young women diagnosed with EC (age less than 50 years) from three major medical institutions were included in this study. More than one-half (524/628, 83.4%) of patients were diagnosed with EC in the age range of 40-49 years, and majority of early-onset EC patients were diagnosed between the ages of 45 and 49 years (368/628, 58.6%, **Supplementary Figure S2**). The clinicopathological features of the training cohort (FAHCQMU cohort, n = 458), and the validation cohort (WCHCQMU & CYMCHC cohorts, n = 170) are shown in **Table 1**. The mean age of onset for early-onset EC was 44.1 ± 5.0 years, and most of the uterine tumors found were histological type I (76.1%), FIGO I (69.3%), and LVSI negative (77.4%). IHC analysis highlighted abnormal P53 expression and dMMR status in 145/628 (23.1%) and 172/628 (27.4%) cases, respectively. Additionally, 10.8% of the cases experienced relapse during the follow-up period. The distribution of clinicopathological characteristics among early-onset EC in training cohort was similar to that of validation cohort (*P* > 0.05).

**Table 1:** Demographics and clinical characteristics of the study population.

Characteristic	Total	Development cohort (n = 458) n (%)/mean (SD)	Validation cohort (n = 170) n (%)/mean (SD)	*P*-value
**Age at diagnosis (years)**	44.1 ± 5.0	44.0 ± 5.0	44.1 ± 4.9	0.846
**BMI (kg/m^2^)**	24.7 ± 3.7	24.8 ± 3.7	24.6 ± 3.5	0.515
**FIGO stage**				0.410
I	435 (69.3)	319 (69.6)	116 (68.2)	
II	100 (15.9)	76 (16.6)	24 (14.1)	
III	93 (14.8)	63 (13.8)	30 (17.7)	
**Histological subtype**				0.475
Type I	478 (76.1)	352 (76.9)	126 (74.1)	
Type II	150 (23.9)	106 (23.1)	44 (25.9)	
**Myometrial invasion**				0.742
< 1/2	452 (72.0)	328 (71.6)	124 (72.9)	
≥ 1/2	176 (28.0)	130 (28.4)	46 (27.1)	
**Cervical involvement**				0.796
No	492 (78.3)	360 (78.6)	132 (77.6)	
Yes	136 (21.7)	98 (21.4)	38 (22.4)	
**LVSI**				0.207
No LVSI	486 (77.4)	360 (78.6)	126 (74.1)	
Focal LVSI	96 (15.3)	63 (13.8)	33 (19.4)	
Substantial LVSI	46 (7.3)	35 (7.6)	11 (6.5)	
**P53 expression**				0.099
Normal	483 (76.9)	360 (78.6)	123 (72.4)	
Abnormal	145 (23.1)	98 (21.4)	47 (27.6)	
**MMR status**				0.753
pMMR	456 (72.6)	331 (72.3)	125 (73.5)	
dMMR	172 (27.4)	127 (27.7)	45 (26.5)	
**Recurrence**				0.864
No	560 (89.2)	409 (89.3)	151 (88.8)	
Yes	68 (10.8)	49 (10.7)	19 (11.2)	
**Adjuvant treatment**				0.563
Follow-up	323 (51.4)	232 (50.7)	91 (53.5)	
Only radiotherapy	185 (29.5)	133 (29.0)	52 (30.6)	
Only chemotherapy	21 (3.3)	15 (3.3)	6 (3.5)	
Chemo-radiotherapy	99 (15.8)	78 (17.0)	21 (12.4)	

LVSI, lymphovascular space invasion; pMMR, proficient mismatch repair; dMMR, deficient mismatch repair.

In the training cohort, a total of 49 patients relapsed, with a median RFS of 51.0 months (range, 3-91); while in the validation cohort, 19 patients relapsed, with a median RFS of 53.0 months (range, 4-90). The recurrence sites were similar between the two cohorts (*P* > 0.05). Detailed information on recurrence characteristics was summarized in **Table 2**.

**Table 2:** Recurrence characteristics of early-onset EC.

Variable	Training cohort (n = 458)	%	Validation cohort (n = 170)	%	*P*-value
**Recurrence**					0.864
No	409	89.3	151	88.8	
Yes	49	10.7	19	11.2	
**Sites of replased**					0.855
Vaginal stump	1	2.0	0	0.0	
Central pelvic region	11	22.4	5	26.3	
Lymph nodes (upper para-aortic)	4	8.2	1	5.3	
Peritoneal metastases	6	12.3	4	21.0	
Metastasis to other organs	27	55.1	9	47.4	
**RFS (months)**					0.820
Median	51.00		53.00		
Mean (± SD)	52.36 (± 17.59)		52.01 (± 14.93)		
Range	3-91		4-90		

RFS, recurrence-free survival; SD, standard deviation.

### 3.2 Independent prognostic factors of RFS

Within the training cohort, both clinicopathologic features and immunohistochemical markers were included in the univariate and multivariate analysis. Multivariate Cox analysis showed that histological type II (HR 2.122, 95% CI 1.119-4.022, *P* = 0.021), FIGO stage III (HR 3.088, 95% CI 1.469-6.491, *P* = 0.003), deep myometrial invasion (HR 2.063, 95% CI 1.036-4.108, *P* = 0.039), substantial LVSI (HR 2.591, 95% CI 1.291-5.198, *P* = 0.007), and abnormal P53 expression (HR 3.350, 95% CI 1.848-6.073, *P* < 0.001), dMMR status (HR 1.863, 95% CI 1.026-3.382, *P* = 0.041) were identified as independent risk factors for RFS in patients with early-onset EC (**Table 3**).

**Table 3:** Factors predicting the RFS of early-onset EC by univariate and multivariate Cox regression analysis.

Variable	Univariate analysis			Multivariate analysis		
	Hazard ratio	95% CI	*P*-value	Hazard ratio	95% CI	*P*-value
**Age at diagnosis (years)** (≥45 vs <45)	1.834	0.961-3.501	0.066	–	–	–
**Histological subtype** (Type II vs Type I)	1.963	1.029-3.745	0.041	2.122	1.119-4.022	0.021
FIGO stage						
I	Ref			Ref		
II	2.444	0.887-6.728	0.084	2.732	1.285-5.808	0.009
III	3.033	1.223-7.524	0.017	3.088	1.469-6.491	0.003
**Myometrial invasion** (≥1/2 vs <1/2)	2.245	1.138-4.426	0.020	2.063	1.036-4.108	0.039
**Cervical involvement** (Yes vs No)	1.080	0.487-2.395	0.851	–	–	–
LVSI						
No LVSI	Ref			Ref		
Focal LVSI	1.282	0.585-2.810	0.535	1.219	0.559-2.655	0.619
Substantial LVSI	2.852	1.401-5.806	0.004	2.591	1.291-5.198	0.007
**P53 expression** (Abnormal vs Normal)	3.761	2.005-7.053	<0.001	3.350	1.848-6.073	<0.001
**MMR status** (dMMR vs pMMR)	1.892	1.042-3.432	0.036	1.863	1.026-3.382	0.041

Ref, reference; CI, confidence interval; HR, hazard ratio; LVSI, lymphovascular space invasion; pMMR, proficient mismatch repair; dMMR, deficient mismatch repair.

### 3.3 Establishing and validation of the nomogram

**Figure 3:**
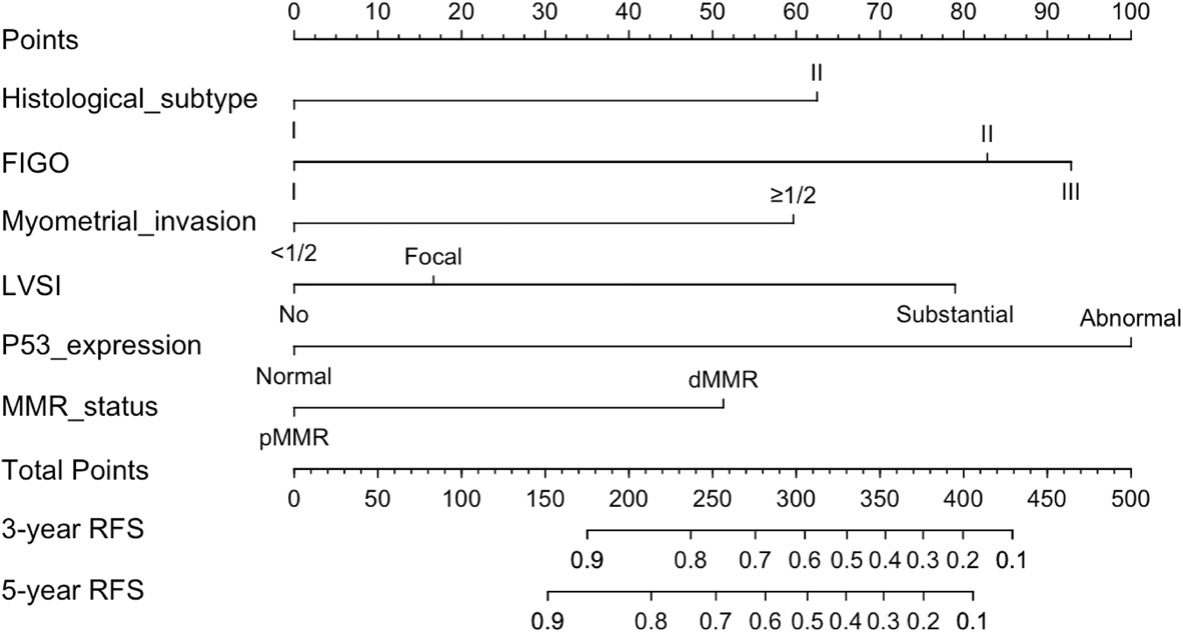
The nomogram was established to predict the 3-, and 5-year RFS of early-onset EC after hysterectomy.

**Figure 4:**
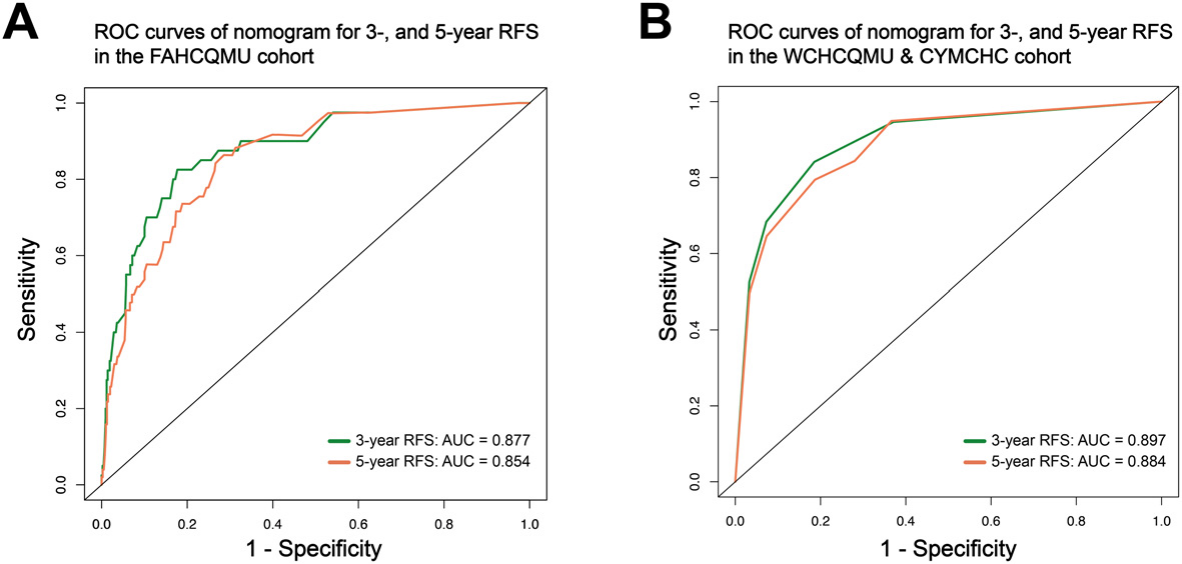
The ROC curve of the nomogram to predict 3-, and 5-year RFS rates in the training cohort **(A)**; and in the validation cohort **(B)**.

**Figure 5:**
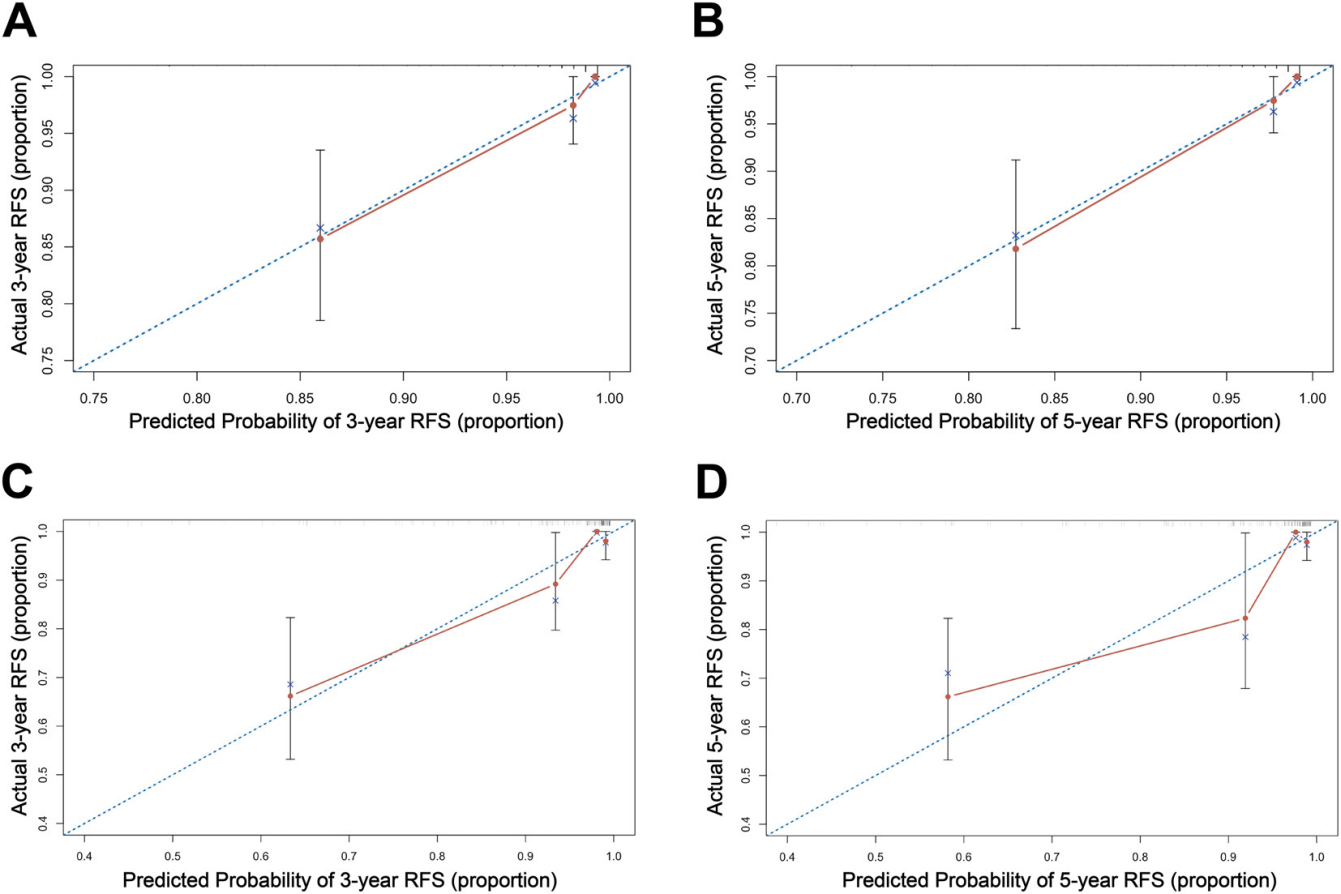
The calibration curve of the nomogram for 3-, and 5-year RFS in the training cohort **(A, B)**; and in the validation cohort **(C, D)**.

Based on the multivariate Cox regression analysis, we constructed a nomogram model for personalized prediction of RFS by calculating each patient’s weighted score (**Figure 3**). According to the degree of contribution of each predictor to the resulting events (RFS), the corresponding points (the first axis) were obtained. Then, the points of each predictor were summed to predict the 3- and 5-year RFS probability of early-onset EC patients. The C-index for nomogram of RFS was 0.844 (95% CI 0.795-0.893) in the training cohort and 0.876 (95% CI 0.807-0.945) in the validation cohort, outperforming the use of clinical parameters and immunohistochemical markers individually (**Supplementary Table S2**). In addition, the ROC curve was utilized to calculate the AUC of the nomogram, revealing that AUC values of both the training cohort and validation cohort were greater than 0.85 (**Figure 4**). The calibration curves revealed an excellent coherence between predicted 3- and 5-year RFS rates and actual RFS rates, indicating the predictive accuracy of the nomogram model (**Figure 5**).

### 3.4 Influence of various variables on the RFS of early-onset EC patients

**Figure 6:**
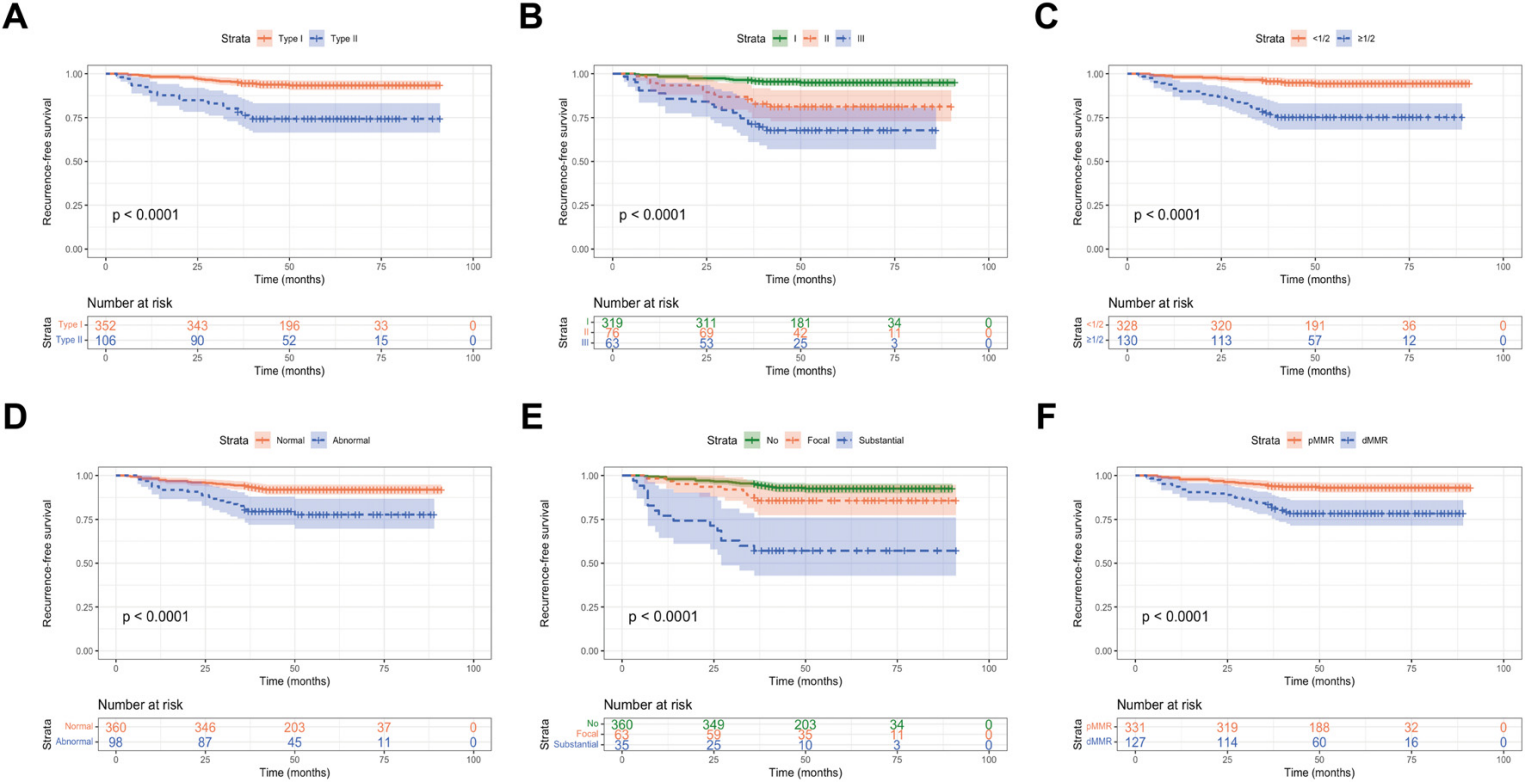
RFS of early-onset EC according to **(A)** histological subtype; **(B)** FIGO stage; **(C)** myometrial invasion; **(D)** P53 expression; **(E)** LVSI; and **(F)** MMR expression.

Kaplan-Meier survival analysis was used to evaluate the RFS among early-onset EC patients in the training set. Young EC patients with histological type II, FIGO stage III, and deep myometrial invasion had a worse RFS. Additionally, in young patients, postoperative pathology showing substantial LVSI, abnormal P53 expression and dMMR status indicated a higher probability of postoperative recurrence and shorter RFS (*P* < 0.001, **Figure 6**).

### 3.5 Performance of the nomogram in stratifying risk

**Figure 7:**
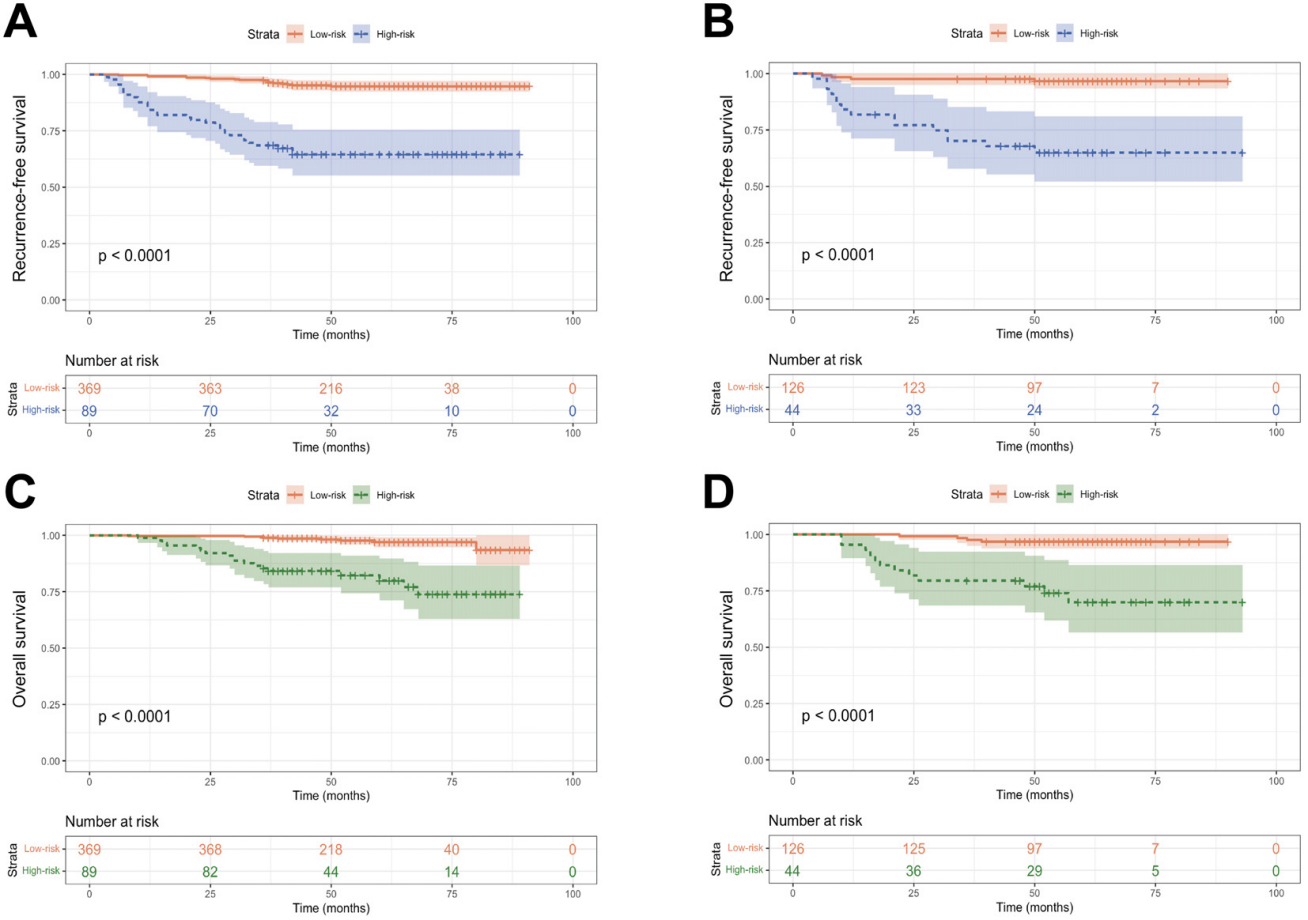
Kaplan-Meier curves of the low-risk groups and high-risk group. **(A, B)** RFS curves of patients with early-onset EC stratified by the risk scores in the training cohort and external validation cohorts; **(C, D)** OS curves of patients with early-onset EC stratified by the risk scores in the training cohort and external validation cohorts.

In order to further validate our prediction model, patients from the training cohort and validation cohort were stratified into low- and high-risk groups based on the nomogram-generated scores (NGS) for RFS. The cut-off value of 249.1 was determined using X-tile (**Supplementary Figure S3**). Kaplan-Meier curves indicated that patients classified as high-risk had significantly worse RFS and OS outcomes compared to those in the low-risk group (*P* < 0.001, **Figure 7**). Furthermore, the 3- and 5-year RFS and OS rates of patients in the high-risk group were much lower than those in the low-risk group in both cohorts (*P* < 0.001, **Table 4**).

**Table 4:** Analysis of survival differences between high- and low-risk groups in training cohort and validation cohort.

Cohort	Group	3-year RFS rate (95% CI)	5-year RFS rate (95% CI)	*P*-value^a^	3-year OS rate (95% CI)	5-year OS rate (95% CI)	*P*-value^b^
Training Cohort (n = 458)	High-risk group (n =89)	68.5% (58.9%-78.1%)	64.5% (54.5%-74.5%)	<0.001	85.4% (78.1%-92.7%)	79.9% (70.7%-89.1%)	<0.001
	Low-risk group (n = 369)	97.3% (95.7%-98.9%)	94.7% (92.3%-97.1%)		98.9% (97.9%-99.8%)	97.3% (95.1%-99.5%)	
Validation Cohort (n = 170)	High-risk group (n = 44)	70.1% (56.6%-83.6%)	65.0% (50.7%-79.3%)	<0.001	79.5% (76.6%-92.8%)	69.9% (44.2%-82.8%)	<0.001
	Low-risk group (n = 126)	97.6% (95.4%-99.8%)	96.6% (93.9%-99.3%)		97.6% (95.4%-99.8%)	96.8% (95.2%-98.4%)	

a Log rank test of RFS.

b Log rank test of OS.

CI, confidence interval.

**Figure 8:**
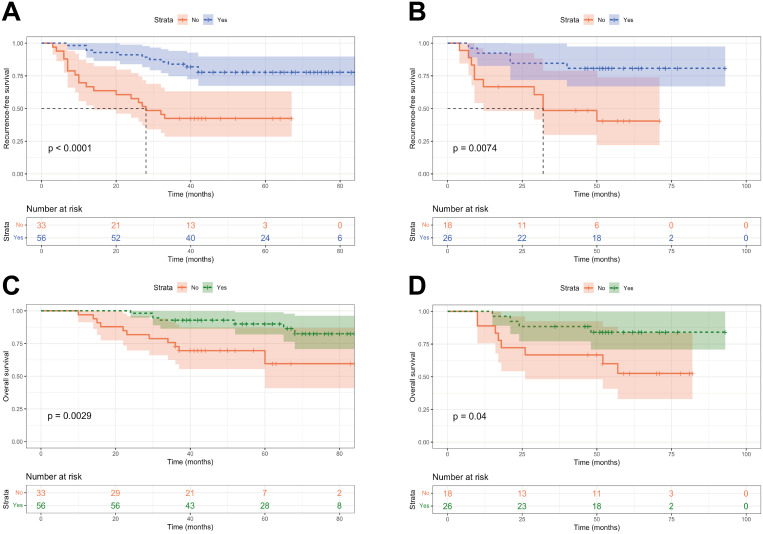
Kaplan-Meier curves of patients with or without adjuvant treatment in high-risk group. **(A, B)** RFS curves of patients with or without adjuvant treatment in high-risk group in the training and validation cohorts; **(C, D)** OS curves of patients with or without adjuvant treatment in high-risk group in the training and validation cohorts.

To further assess the potential of risk stratification in guiding postoperative adjuvant therapy, we compared the two groups to identify individuals who could benefit from adjuvant therapy. In the low-risk group, there was no significant difference in RFS and OS between patients who received adjuvant therapy and those who did not (*P* > 0.05, **Supplementary Figure S4**). Among patients in the high-risk group, to our surprise, individuals who received postoperative adjuvant therapy had significantly better RFS and OS than those who received no adjuvant therapy (*P* < 0.05, **Figure 8**).

## 4 Discussion

The increasing prevalence of early-onset EC has resulted in the development of a distinct and unique subset among EC patients, characterized by differences in disease-free survival rates and tumor characteristics (22, 23). Our previous studies have compared the RFS of young EC patients who underwent various fertility-sparing treatments and hysterectomy, showing that surgical intervention could improve the prognosis of early-onset EC patients. Consequently, the present study focuses primarily on early-onset EC patients receiving surgical treatment (**Supplementary Table S3**; **Supplementary Figures S5**, **S6**). In our study, the initial diagnosis age of early-onset EC is concentrated between 45 and 49 years. Therefore, appropriately screening EC patients aged 45-49 years may help in the early detection and diagnosis of EC in individuals. Although previous studies have indicated a favorable prognosis for early-onset EC, our research still reports a 10.8% postoperative recurrence rate in early-onset EC patients. Therefore, accurate and effective prediction of recurrence and prognosis in this subgroup contributes to guiding clinical practice and treatment decisions.

In this study, we identified six independent risk factors for predicting RFS of early-onset EC patients. These factors include histological subtype, FIGO stage, myometrial invasion, LVSI, P53 expression, and MMR expression. Based on these clinical parameters and immunohistochemical markers, we developed a robust nomogram to predict the 3- and 5-year RFS rates of early-onset EC patients. By utilizing weighted scores, early-onset EC patients can be divided into high- and low-risk group, providing accurate RFS for each patient and guiding clinical treatment.

Histological subtypes are one of the key features of the revised 2023 FIGO staging, which includes two different types. Type I is composed of low-grade (grades 1 and 2) endometrioid adenocarcinoma (EEC), resulting from atypical hyperplasia due to chronic estrogen stimulation, and typically exhibit non-aggressive characteristics. Type II comprises high-grade (grade 3) EEC and non-endometrioid cancers, which often have a worse prognosis (2). Given the poor prognosis of advanced EEC and non-endometrioid EC, as well as the significant association between histological subtypes and recurrence in EC patients, we incorporated histological subtypes as a variable in this study. Through multivariable Cox regression analysis, histological type II was identified as an independent risk factor for early-onset EC. Additionally, early-onset EC patients with high-grade EEC or non-endometrioid cancer have a poorer RFS compared to low-grade EEC. Recent studies suggested different histological subtypes have distinct molecular characteristics and precursor lesions, playing essential role in risk stratification of EC, which is consistent with our research (24).

In this study, we demonstrated that LVSI is a significant prognostic variable, and based on NGS, LVSI is identified as one of the primary factors influencing RFS in early-onset EC. Previous studies have confirmed that LVSI is an independent negative prognostic factor for recurrence, which is consistent with our findings (25). A pooled analysis of the PORTEC 1 and 2 trials revealed that substantial LVSI is the strongest predictor for pelvic regional recurrence and distant metastasis, indicating that early-stage patients with substantial LVSI should consider receiving adjuvant EBRT and/or chemotherapy (15). However, most studies have explored the impact of LVSI on recurrence and survival prognosis in EC patients. In comparison to these studies, this research focuses on the significant role of LVSI in recurrence among early-onset EC patients. Our study also indicates that early-onset EC patients with substantial LVSI have shorter RFS and worse prognosis compared to no/focal LVSI patients. Furthermore, our study demonstrates that myometrial invasion ≥ 50% is another independent risk factor for recurrence in early-onset EC. For the assessment of prognosis in early-onset EC, account needs to be taken of deep myometrial invasion. Therefore, combining histological subtypes, FIGO staging, myometrial invasion, and LVSI can significantly enhance the predictive ability of RFS in early-onset EC patients.

The clinical management of high-risk and advanced EC is transitioning from histological subtype to molecular classifications. However, the high cost and technical requirements of genetic testing limit its widespread use as a routine diagnostic tool in many developing regions. By identifying the molecular characteristics of tumors, immunohistochemistry can serve as a good transition and complement to genetic testing, providing clinicians with a more comprehensive understanding of the tumor. Current studies have not specifically reported on the impact of immunohistochemical markers on the recurrence of early-onset EC patients. In fact, relying solely on clinical features cannot effectively assess the prognosis of early-onset EC patients. Therefore, further incorporating immunohistochemical molecular markers for a comprehensive evaluation of the prognosis of early-onset EC patients is of significant importance. Our study analyzed commonly used immunohistochemical markers and ultimately identified abnormal P53 expression and complete loss of MMR protein expression as risk factors for recurrence in early-onset EC patients. MMR deficiency or MSI is common in EC, affecting 20% to 40% of patients, and impacting risk stratification, treatment decisions, and Lynch syndrome (LS) screening (26–28). With increasing emphasis on molecular classification in EC, MMR status has emerged as a crucial biomarker in ongoing clinical trials (GINECOEN105b, ClinicalTrials.gov identifier, NCT05201547; RAINBO, ClinicalTrials.gov identifier NCT05255653). While the value of MMR status in assessing recurrence and prognosis in EC patients remains controversial, our study indicated that MMR status is one of the independent predictors of recurrence in early-onset EC patients. In addition to the potential value of MMR status in refining risk stratification, this result may be partly related to the higher proportion of LS carriers among early-onset EC patients. Therefore, screening for LS in young populations or specific high-risk groups is of paramount importance (29, 30). The aim of screening is to detect atypical hyperplasia or EC at the earliest stage to improve chances of cure and minimize treatment-related morbidity. Reducing mortality from this disease and enhancing cancer screening and prevention among family members is challenging and requires further high-quality research. Aberrant P53 protein expression was typically associated with aggressive histology and clinical course (31, 32). For clinical outcomes prediction, a previous study showed EC patients with *TP53* missense mutations and P53 protein over-expression exhibited comparable impacts on progression-free survival and overall survival. Furthermore, the concordance between *TP53* next-generation sequencing (NGS) and P53 IHC was 88%; this percentage increased to 92% when cases with *TP53* mutations accompanied by *POLE*mut or dMMR were excluded (17).

Noteworthy, factors such as age and cervical stromal invasion were not identified as predictors for RFS. This could be explained by the “collinearity” among different risk factors and the specific population included in present study. Hence, we established a nomogram based on above six readily available variables to assist clinicians in assessing patients’ RFS. Additionally, we further stratified early-onset EC patients based on NGS for risk assessment. It is worth noting that adjuvant therapy reduced loco-regional recurrence in high-risk cases and demonstrated significantly better RFS and OS compared to those who received no adjuvant therapy. In the low-risk group, RFS and OS of patients who received postoperative adjuvant therapy were comparable to those who did not, suggesting that patients in the high-risk group may require more proactive postoperative management than those in the low-risk group. Therefore, risk stratification for early-onset EC patients is essential for the risk-benefit analysis of adjuvant therapy in the context of patient individualization. The nomogram integrating multiple clinical variables and immunohistochemical molecular markers demonstrates superior predictive performance in assessing the RFS of early-onset EC patients compared to using clinical variables and immunohistochemical markers separately.

In light of the above, there remains a significant opportunity and unmet need for novel diagnosis approaches or prediction models in early-onset EC to improve their prognosis. To our knowledge, this is the first multicenter study that integrates clinicopathological parameters and immunohistochemical markers to analyze risk factors for RFS in early-onset EC patients and develop a predictive model, although there are still limitations. One limitation of our study is the inability to include *POLE* mutation status in the analysis due to small number of such cases. This resulted in a small portion of patients harboring multiple molecular subtypes, such as *POLE*mut and P53abn, being classified as the P53abn subtype, potentially contributing to an overestimation of the risk of recurrence. In fact, previous studies have shown that patients with *POLE*mut accounted for a relatively low proportion (about 5-8%) among the TCGA molecular subtypes of all EC patients, while the proportion of patients harboring multiple molecular subtypes simultaneously is even lower, approximately 3%-5% (33–35). Therefore, the model established in this study overestimates the risk of recurrence in high-risk patients to a relatively low extent. Furthermore, another limitation of this study is its reliance on retrospective analysis. Due to the early diagnosis and low post-surgery recurrence rate of most early-onset EC patients, the study included a small number of recurrent cases relative to the prognostic factors involved in the multivariate analysis, which may cause statistical bias to a certain extent. Therefore, necessitating further validation of the universality of this model through further prospective clinical trials.

## 5 Conclusion

In summary, this study developed and verified a highly accurate nomogram for the prediction of RFS in early-onset EC patients after surgery. This novel risk stratification system can serve as an important supplement to FIGO staging and molecular subtyping in predicting RFS of early-onset EC patients, providing personalized treatment guidance for this specific subgroup and improving clinical decision-making.

## Data Availability

The datasets used and/or analyzed during the current study are available from the corresponding author on reasonable request. Requests to access the datasets should be directed to Rui Yuan, yrui96@hospital.cqmu.edu.cn.

## References

[1] Sung H Ferlay J Siegel RL Laversanne M Soerjomataram I Jemal A. Global cancer statistics 2020: GLOBOCAN estimates of incidence and mortality worldwide for 36 cancers in 185 countries. CA Cancer J Clin. (2021) 71:209–49. doi: 10.3322/caac.21660 33538338

[2] Berek JS Matias-Guiu X Creutzberg C Fotopoulou C Gaffney D Kehoe S. FIGO staging of endometrial cancer: 2023. J Gynecol Oncol. (2023) 34:e85. doi: 10.3802/jgo.2023.34.e85 37593813 PMC10482588

[3] Colombo N Preti E Landoni F Carinelli S Colombo A Marini C. Endometrial cancer: ESMO Clinical Practice Guidelines for diagnosis, treatment and follow-up. Ann Oncol. (2011) 22 Suppl 6:vi35–39. doi: 10.1093/annonc/mdr374 21908501

[4] Choi J Holowatyj AN Du M Chen Z Wen W Schultz N. Distinct genomic landscapes in early-Onset and late-Onset endometrial cancer. JCO Precis Oncol. (2022) 6:e2100401. doi: 10.1200/PO.21.00401 35108035 PMC8820918

[5] Walsh MD Cummings MC Buchanan DD Dambacher WM Arnold S McKeone D. Molecular, pathologic, and clinical features of early-onset endometrial cancer: identifying presumptive Lynch syndrome patients. Clin Cancer Res. (2008) 14:1692–700. doi: 10.1158/1078-0432.CCR-07-1849 18310315

[6] Crissman JD Azoury RS Barnes AE Schellhas HF. Endometrial carcinoma in women 40 years of age or younger. Obstet Gynecol. (1981) 57:699–704. 7015203

[7] Banz-Jansen C Helweg LP Kaltschmidt B. Endometrial cancer stem cells: where do we stand and where should we go? Int J Mol Sci. (2022) 23:3412. doi: 10.3390/ijms23063412 35328833 PMC8955970

[8] Kandoth C Schultz N Cherniack AD Akbani R Liu Y Shen H. Integrated genomic characterization of endometrial carcinoma. Nature. (2013) 497:67–73. doi: 10.1038/nature12113 23636398 PMC3704730

[9] Hruda M Sehnal B Halaška MJ Drozenová J Robová H Pichlík T. New staging of endometrial carcinoma - FIGO 2023. Ceska Gynekol. (2024) 89:120–7. doi: 10.48095/cccg2024120 38704224

[10] Colombo N Creutzberg C Amant F Bosse T González-Martín A Ledermann J. ESMO-ESGO-ESTRO Consensus Conference on Endometrial Cancer: diagnosis, treatment and follow-up. Ann Oncol. (2016) 27:16–41. doi: 10.1093/annonc/mdv484 26634381

[11] Bendifallah S Ilenko A Daraï E. High risk endometrial cancer: Clues towards a revision of the therapeutic paradigm. J Gynecol Obstet Hum Reprod. (2019) 48:863–71. doi: 10.1016/j.jogoh.2019.06.003 31176047

[12] Concin N Creutzberg CL Vergote I Cibula D Mirza MR Marnitz S. ESGO/ESTRO/ESP Guidelines for the management of patients with endometrial carcinoma. Virchows Arch. (2021) 478:153–90. doi: 10.1007/s00428-020-03007-z 33604759

[13] Jiang P Wang J Gong C Yi Q Zhu M Hu Z. A nomogram model for predicting recurrence of stage I-III endometrial cancer based on inflammation-immunity-nutrition score (IINS) and traditional classical predictors. J Inflammation Res. (2022) 15:3021–37. doi: 10.2147/JIR.S362166 PMC9135581 35645577

[14] Fujimoto T Nanjyo H Fukuda J Nakamura A Mizunuma H Yaegashi N. Endometrioid uterine cancer: histopathological risk factors of local and distant recurrence. Gynecol Oncol. (2009) 112:342–7. doi: 10.1016/j.ygyno.2008.10.019 19062082

[15] Bosse T Peters EE Creutzberg CL Jürgenliemk-Schulz IM Jobsen JJ Mens JW. Substantial lymph-vascular space invasion (LVSI) is a significant risk factor for recurrence in endometrial cancer–A pooled analysis of PORTEC 1 and 2 trials. Eur J Cancer. (2015) 51:1742–50. doi: 10.1016/j.ejca.2015.05.015 26049688

[16] Camp RL Dolled-Filhart M Rimm DL. X-tile: a new bio-informatics tool for biomarker assessment and outcome-based cut-point optimization. Clin Cancer Res. (2004) 10:7252–9. doi: 10.1158/1078-0432.CCR-04-0713 15534099

[17] Thiel KW Devor EJ Filiaci VL Mutch D Moxley K Alvarez Secord A. TP53 sequencing and p53 immunohistochemistry predict outcomes when bevacizumab is added to frontline chemotherapy in endometrial cancer: an NRG oncology/gynecologic oncology group study. J Clin Oncol. (2022) 40:3289–300. doi: 10.1200/JCO.21.02506 PMC9553389 35658479

[18] Zong L Mo S Sun Z Lu Z Chen J Yu S. Incorporating molecular classification when stratifying the survival risk of patients with high-Grade endometrial carcinomas. J Clin Med. (2023) 12:530. doi: 10.3390/jcm12020530 36675462 PMC9866413

[19] Antill Y Kok PS Robledo K Yip S Cummins M Smith D. Clinical activity of durvalumab for patients with advanced mismatch repair-deficient and repair-proficient endometrial cancer. A nonrandomized phase 2 Clin trial J Immunother Cancer. (2021) 9:e002255. doi: 10.1136/jitc-2020-002255 PMC8190057 34103352

[20] Ukkola I Nummela P Pasanen A Kero M Lepistö A Kytölä S. Detection of microsatellite instability with Idylla MSI assay in colorectal and endometrial cancer. Virchows Arch. (2021) 479:471–9. doi: 10.1007/s00428-021-03082-w PMC8448708 33755781

[21] Zheng Y Jiang P Tu Y Huang Y Wang J Gou S. Incidence, risk factors, and a prognostic nomogram for distant metastasis in endometrial cancer: A SEER-based study. Int J Gynaecol Obstet. (2024) 165:655–65. doi: 10.1002/ijgo.15264 38010285

[22] Abdol Manap N Ng BK Phon SE Abdul Karim AK Lim PS Fadhil M. Endometrial cancer in pre-menopausal women and younger: risk factors and outcome. Int J Environ Res Public Health. (2022) 19:9059. doi: 10.3390/ijerph19159059 35897440 PMC9330568

[23] Semaan A Ali-Fehmi R Munkarah AR Bandyopadhyay S Morris RT Rizk S. Clinical/pathologic features and patient outcome in early onset endometrial carcinoma: a population based analysis and an institutional perspective from the Detroit metropolitan area, Michigan. Gynecol Oncol. (2012) 124:265–9. doi: 10.1016/j.ygyno.2011.09.027 22044605

[24] Barlin JN Soslow RA Lutz M Zhou QC St Clair CM Leitao MM Jr. Redefining stage I endometrial cancer: incorporating histology, a binary grading system, myometrial invasion, and lymph node assessment. Int J Gynecol Cancer. (2013) 23:1620–8. doi: 10.1097/IGC.0b013e3182a5055e PMC4405774 24126219

[25] Stålberg K Bjurberg M Borgfeldt C Carlson J Dahm-Kähler P Flöter-Rådestad A. Lymphovascular space invasion as a predictive factor for lymph node metastases and survival in endometrioid endometrial cancer - a Swedish Gynecologic Cancer Group (SweGCG) study. Acta Oncol. (2019) 58:1628–33. doi: 10.1080/0284186X.2019.1643036 31373248

[26] Backes FJ Haag J Cosgrove CM Suarez A Cohn DE Goodfellow PJ. Mismatch repair deficiency identifies patients with high-intermediate-risk (HIR) endometrioid endometrial cancer at the highest risk of recurrence: A prognostic biomarker. Cancer. (2019) 125:398–405. doi: 10.1002/cncr.31901 30561762

[27] Egoavil C Alenda C Castillejo A Paya A Peiro G Sánchez-Heras AB. Prevalence of Lynch syndrome among patients with newly diagnosed endometrial cancers. PloS One. (2013) 8:e79737. doi: 10.1371/journal.pone.0079737 24244552 PMC3820559

[28] Makker V Colombo N Casado Herráez A Santin AD Colomba E Miller DS. Lenvatinib plus pembrolizumab for advanced endometrial cancer. N Engl J Med. (2022) 386:437–48. doi: 10.1056/NEJMoa2108330 PMC11651366 35045221

[29] Riedinger CJ Esnakula A Haight PJ Suarez AA Chen W Gillespie J. Characterization of mismatch-repair/microsatellite instability-discordant endometrial cancers. Cancer. (2024) 130:385–99. doi: 10.1002/cncr.35030 PMC10843110 37751191

[30] Stelloo E Jansen AML Osse EM Nout RA Creutzberg CL Ruano D. Practical guidance for mismatch repair-deficiency testing in endometrial cancer. Ann Oncol. (2017) 28:96–102. doi: 10.1093/annonc/mdw542 27742654

[31] Albitar L Carter MB Davies S Leslie KK. Consequences of the loss of p53, RB1, and PTEN: relationship to gefitinib resistance in endometrial cancer. Gynecol Oncol. (2007) 106:94–104. doi: 10.1016/j.ygyno.2007.03.006 17490733

[32] Brachova P Mueting SR Devor EJ Leslie KK. Oncomorphic TP53 mutations in gynecologic cancers lose the normal protein: protein interactions with the microRNA microprocessing complex. J Cancer Ther. (2014) 5:506–16. doi: 10.4236/jct.2014.56058 PMC4203685 25339994

[33] Kommoss S McConechy MK Kommoss F Leung S Bunz A Magrill J. Final validation of the ProMisE molecular classifier for endometrial carcinoma in a large population-based case series. Ann Oncol. (2018) 29:1180–8. doi: 10.1093/annonc/mdy058 29432521

[34] León-Castillo A Gilvazquez E Nout R Smit VT McAlpine JN McConechy M. Clinicopathological and molecular characterisation of 'multiple-classifier' endometrial carcinomas. J Pathol. (2020) 250:312–22. doi: 10.1002/path.5373 PMC7065184 31829447

[35] Raffone A Travaglino A Mascolo M Carbone L Guida M Insabato L. TCGA molecular groups of endometrial cancer: Pooled data about prognosis. Gynecol Oncol. (2019) 155:374–83. doi: 10.1016/j.ygyno.2019.08.019 31472940

