# Supplementary material for: A nomogram model to predict recurrence of early-onset endometrial cancer after resection based on clinical parameters and immunohistochemical markers: a multi-institutional study
Source: Front Oncol. 2024 Nov 11;14:1442489. doi: 10.3389/fonc.2024.1442489 (PMC11586258; doi:10.3389/fonc.2024.1442489)
Supplement: Supplementary file 1 [file DataSheet1.docx]

**Supplementary Materials**

**Content**

Supplementary [Figure 1 The optimal thresholds for early-onset EC patients age were assessed by X-tile..................................................................................................................................................](#_Toc28909)2Supplementary [Figure 2 The age distribution of early-onset EC patients in three medical centers...](#_Toc3135)

[...........................................................................................................................................................2](#_Toc3135)

Supplementary [Figure 3 Cut-off values calculated by X-tile software (A) and (B)..........................3](#_Toc3135)

Supplementary [Figure 4 Kaplan-Meier survival curve of patients with or without adjuvant treatment in low-risk group in two cohorts.......................................................................................3](#_Toc3135)

Supplementary Table 1 Three-tiered approach for lymph vascular space invasion...........................

..........................................................................................................................................................4

Supplementary Table 2 C-index of different risk stratification systems for RFS in the training and validation set.....................................................................................................................................4

Supplementary Table 3 Baseline clinical characteristics and treatment outcomes in women undergoing fertility-sparing treatment of EC....................................................................................5

Supplementary [Figure 5](#_Toc3135) Kaplan-Meier curve of RFS in women with well-differentiated EC stratified by different conservative treatment...................................................................................6

Supplementary [Figure 6](#_Toc3135) Kaplan-Meier curve of RFS in patients with early-onset EC stratified by surgical treatment and conservative treatment.................................................................................6

**
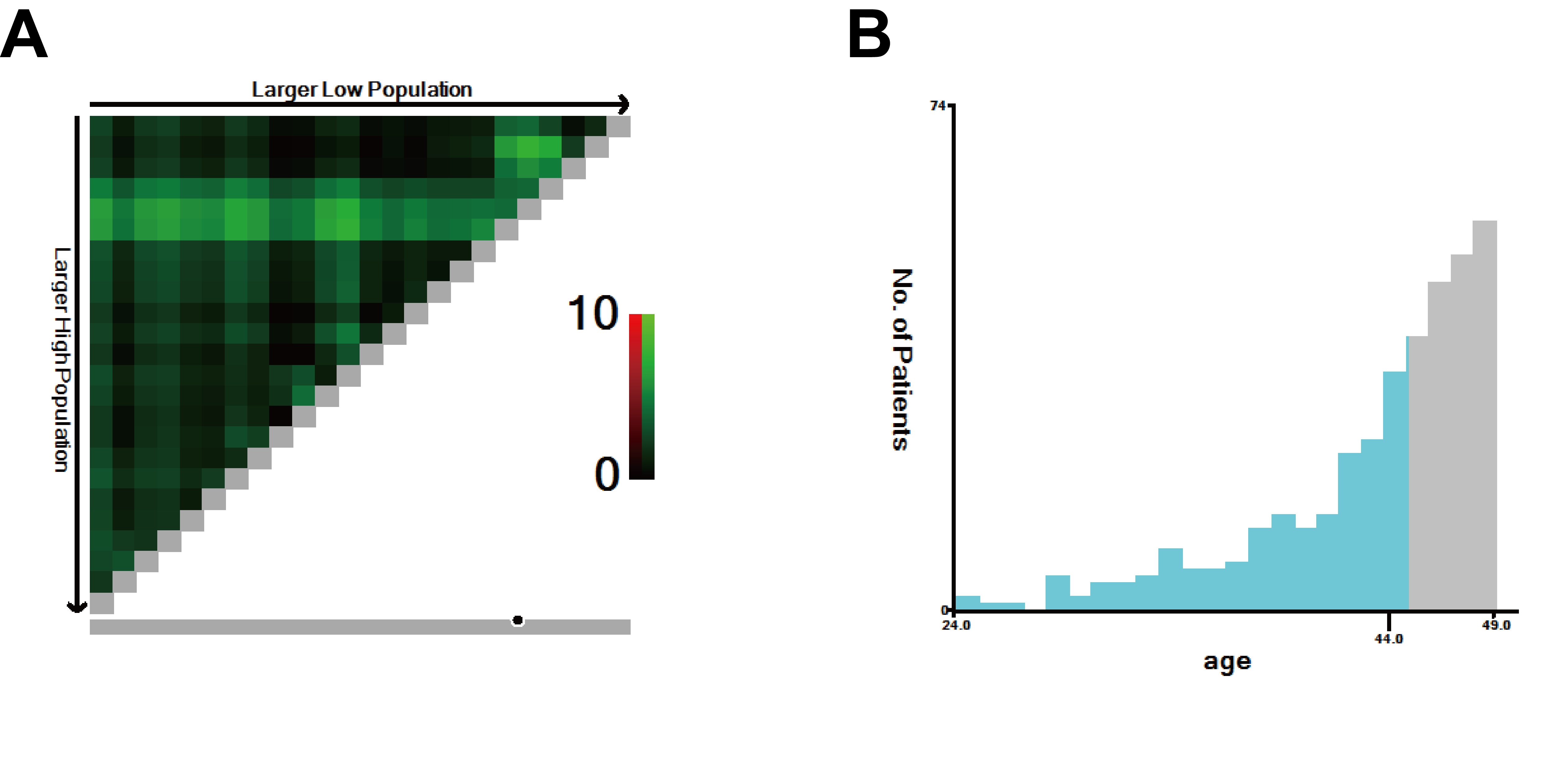
**

**Supplementary Figure 1.** The optimal thresholds for early-onset EC patients age were assessed by X-tile. In accordance with the optimal cut-off value, the age was divided into < 45 years and ≥ 45 years.


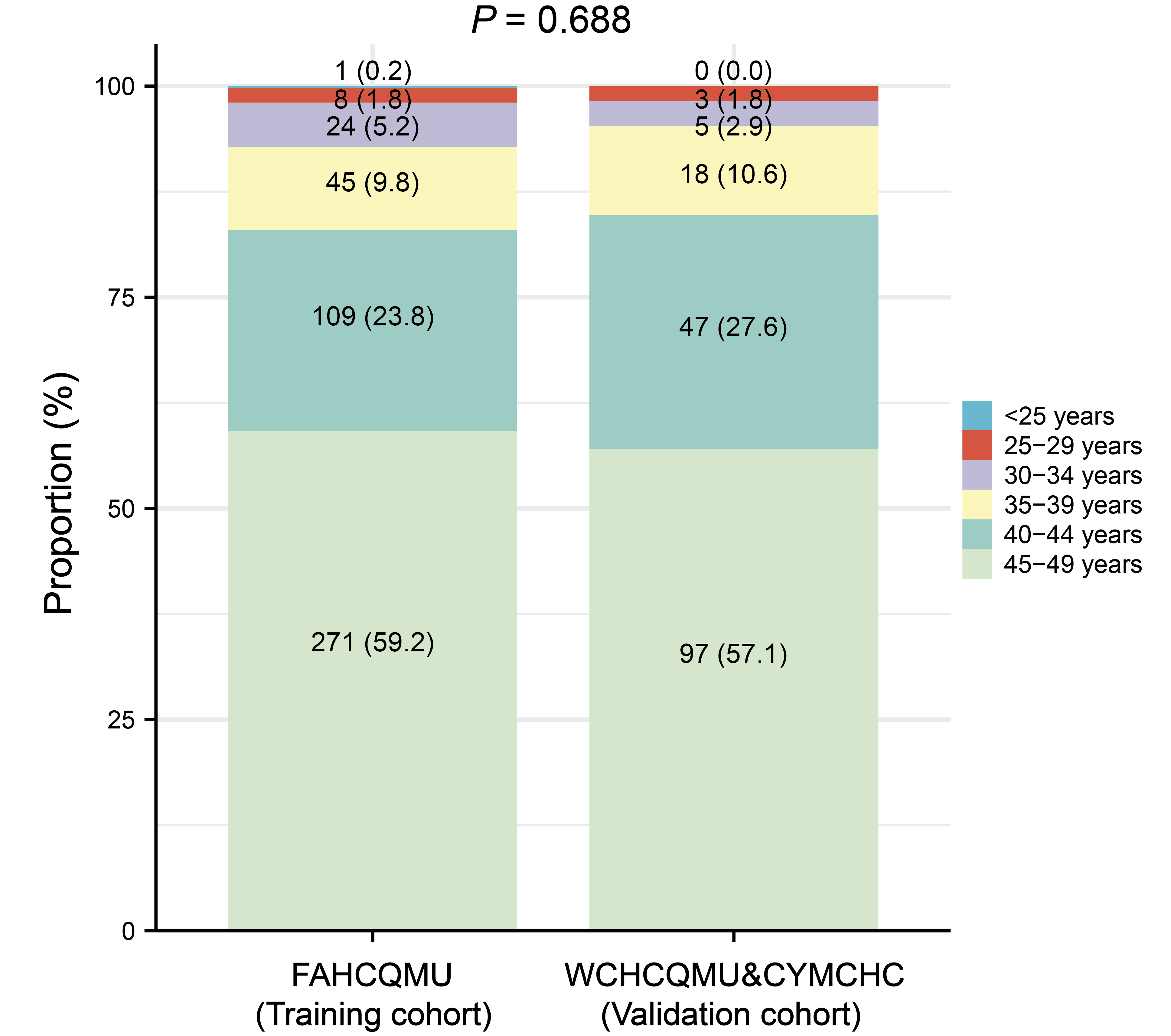


**Supplementary Figure 2.** The age distribution of early-onset EC patients in three medical centers. The mean age of onset of early-onset EC was 44.1 years. The age of patients with early-onset EC is concentrated between 40 and 49 years old, accounting for 83.4% of all early-onset patients. Data are presented as number (percentage).

**
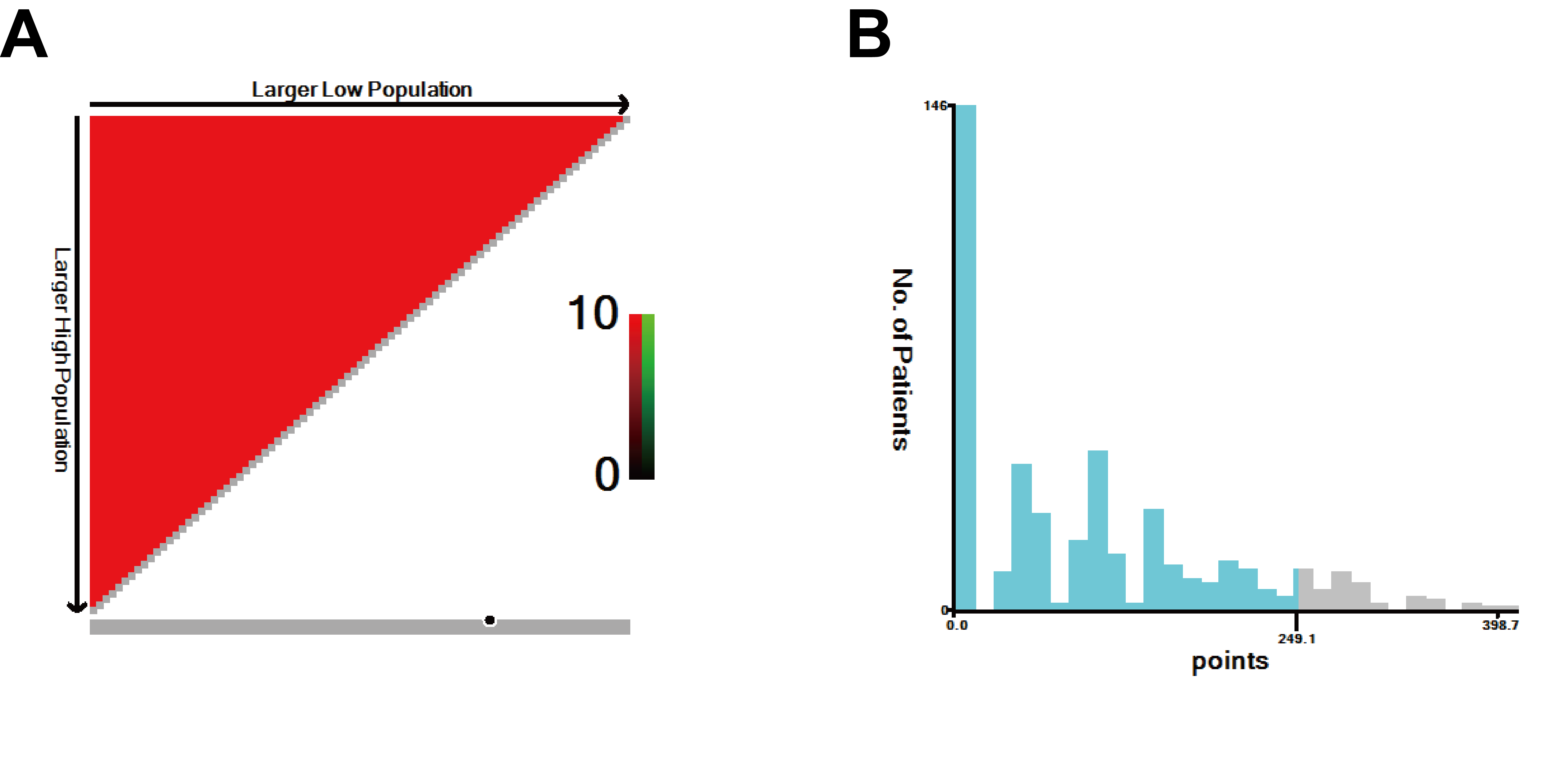
**

**Supplementary Figure 3.** Cut-off values calculated by X-tile software (A) and (B). The determined cut-off value was 249.1, categorizing early-onset EC patients into low-risk group (score < 249.1) and high-risk group (score ≥ 249.1).


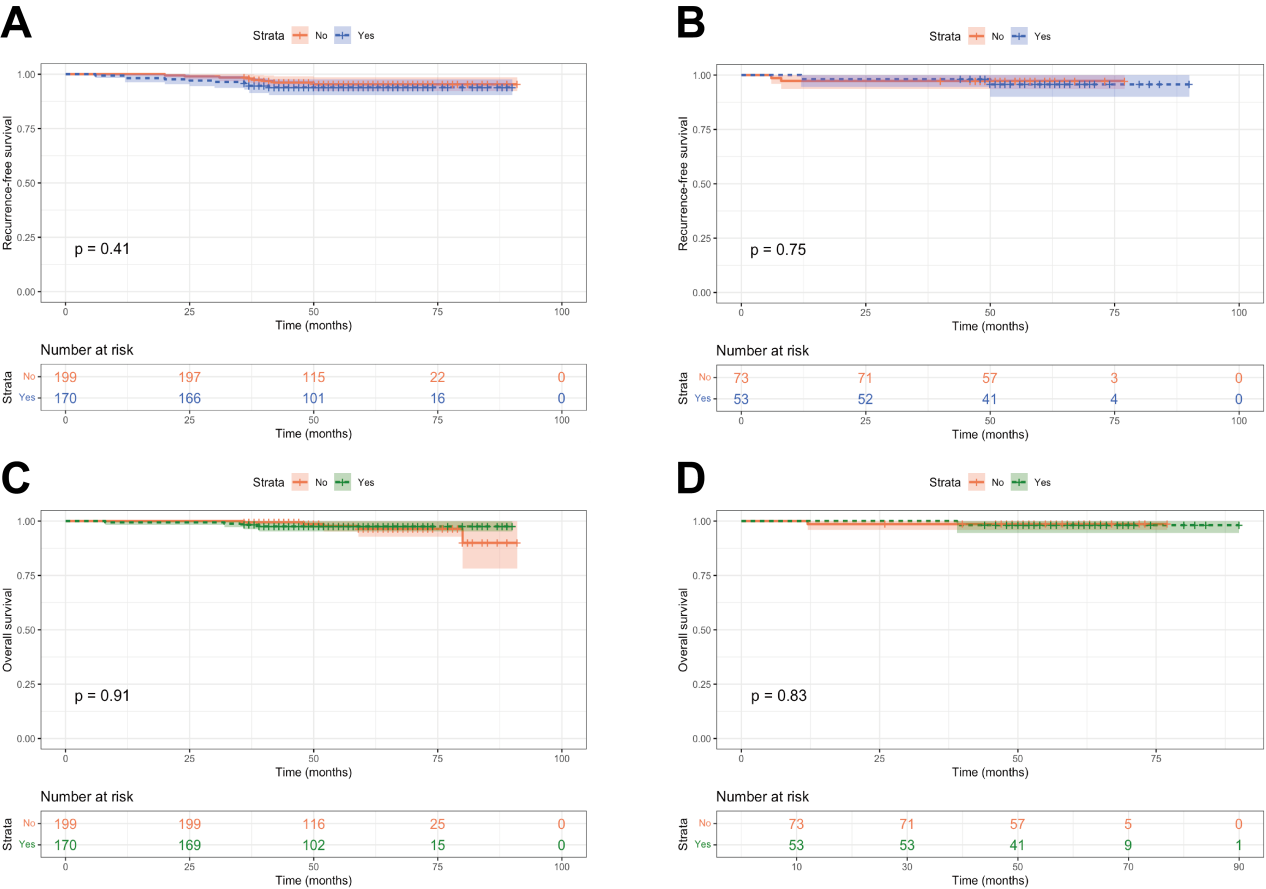


**Supplementary Figure 4.** Kaplan-Meier survival curve of patients with or without adjuvant treatment in low-risk group in two cohorts. (A) RFS curve of patients with or without adjuvant treatment in low-risk group in training cohort. (B) RFS curve of patients with or without adjuvant treatment in validation cohort. (C) OS curve of patients with or without adjuvant treatment in training cohort. (D) OS curve of patients with or without adjuvant treatment in validation cohort.

**Supplementary Table 1.** Three-tiered approach for lymph vascular space invasion

| *Three-tiered approach* | Description^*^ |
| --- | --- |
| No LVSI | Definition not met |
| Focal LVSI | A single focus of LVSI was recognised around a tumor |
| Substantial LVSI | The diffuse or multifocal arrangement of LVSI,  or the presence of tumor cell in 5 or more lymphovascular spaces, was recognised around the tumor |

^*^Definition of LVSI: the presence of tumor cells in a space lined by endothelial cells outside the immediate invasive border.

**Supplementary Table 2.** C-index of different risk stratification systems for RFS in the training and validation set.

| **Risk stratification systems** | **Training set** | | **Validation set** | |
| --- | --- | --- | --- | --- |
|  | **C-index** | **95% CI** | **C-index** | **95% CI** |
| Clinical parameters | 0.805 | 0.748-0.862 | 0.844 | 0.779-0.909 |
| Immunohistochemical markers | 0.706 | 0.637-0.775 | 0.695 | 0.585-0.805 |
| FIGO stage | 0.726 | 0.657-0.795 | 0.758 | 0.662- 0.854 |
| Nomogram model | 0.844 | 0.795-0.893 | 0.876 | 0.807-0.945 |

**Abbreviations:** C-index, concordance index; CI, confidence interval; clinical parameters include histological type, LVSI, and FIGO stage; immunohistochemical markers include P53 and MMR.

**Supplementary Table 3.** Baseline clinical characteristics and treatment outcomes in women undergoing fertility-sparing treatment of EC^a^.

|  | All patients (n = 94) |
| --- | --- |
| Age at diagnosis in years, median (IQR) | 31.0 (28.0-38.0) |
| BMI, median (IQR) | 26.5 (17.2-34.6) |
| Diabetes mellitus | 11 (11.7) |
| PCOS | 12 (12.8) |
| Lymph syndrome |  |
| No | 82 (87.2) |
| Yes | 8 (8.5) |
| Unknown | 4 (4.3) |
| Treatment^b^ |  |
| MPA | 51 (54.3) |
| MA | 20 (21.3) |
| Progestin and LNG-IUD | 10 (10.6) |
| Others^c^ | 13 (13.8) |
| Follow-up time in months, median (IQR) | 33.9 (16.8-82.4) |
| Recurrence (%) | 27 (28.7) |
| Time to recurrence, median (IQR) | 15.0 (7.0-64.2) |

**Note:** a, well-differentiated (grade 1) endometrioid endometrial carcinoma, disease thought to be limited to the endometrium based on magnetic resonance imaging or ultrasound, absence of suspicious or metastatic disease on imaging, and patients have no contraindications to drug therapy or pregnancy and are fully informed; b, MPA, 250-600 mg/d, MA, 160-320 mg/d, treatment for 6 months, progestin and LNG-IUD, placement of LNG-IUS in the uterine cavity and oral administration of MPA (500 mg/d). Discontinue high-dose progestin after the lesion disappears. The duration of LNG-IUS use is determined based on the patient's recent fertility requirements; c, other fertility-preserving treatment included hysteroscopic local lesion resection surgery, combination therapy with LNG-IUS and GnRH-a (3.6 mg/3.75 mg, administered subcutaneously once every 28 days, et al.

**Abbreviations:** PCOS, polycystic ovary syndrome; IQR, interquartile range; LNG-IUD, levonorgestrel-releasing intrauterine device; MPA, medroxyprogesterone acetate; MA, megestrol acetate; GnRH-a, gonadotropin-releasing hormone agonists.


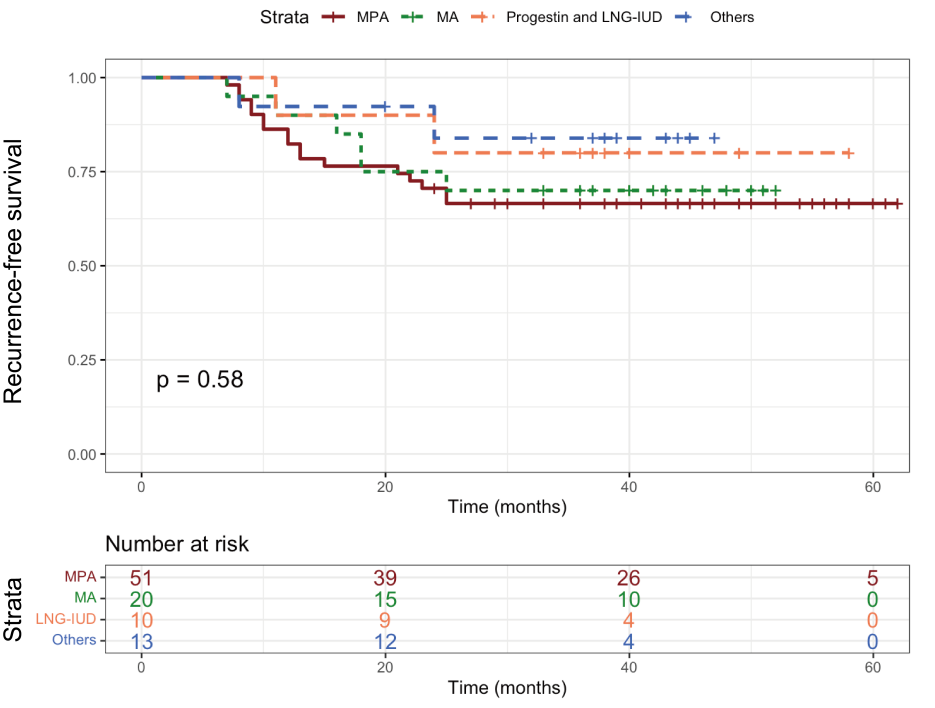


**Supplementary Figure 5.** Kaplan-Meier curve of RFS in women with well-differentiated EC stratified by different conservative treatment. MPA, medroxyprogesterone acetate; MA, megestrol acetate; LNG-IUD, levonorgestrel-releasing intrauterine device; Others, hysteroscopic local lesion resection surgery, combination therapy with LNG-IUS and GnRH-a.


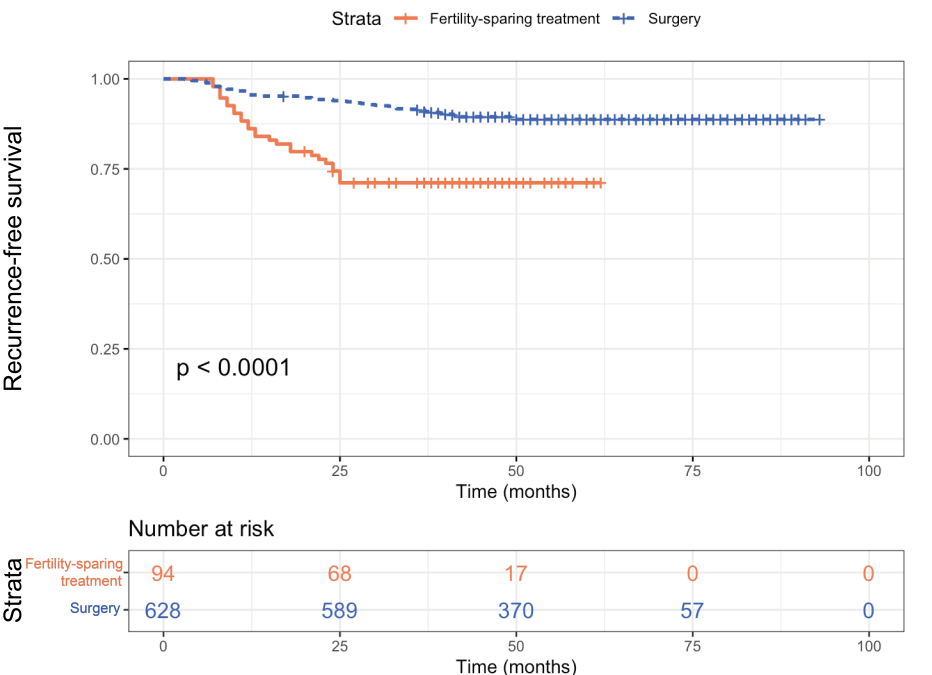


**Supplementary Figure 6.** Kaplan-Meier curve of RFS in patients with early-onset EC stratified by surgical treatment and conservative treatment.
